# Service User Experiences and Perspectives of Social Prescribing Services for Mental Health

**DOI:** 10.1007/s10597-025-01534-0

**Published:** 2025-10-21

**Authors:** Matthew Cooper, Darren Flynn, Jason Scott, Kirsten Ashley, Leah Avery

**Affiliations:** 1 Newcastle University, Newcastle upon Tyne, United Kingdom; 2 National Institute for Health and Care Research Newcastle Patient Safety Research Collaborative, Newcastle upon Tyne, United Kingdom; 3 Department of Nursing, Midwifery and Health, Faculty of Health and Life Sciences, Northumbria University, Newcastle upon Tyne, United Kingdom; 4 School of Health and Life Sciences, Teesside University, Middlesbrough, United Kingdom

## Abstract

To explore service user experiences and perspectives of social prescribing for mental health and to develop theoretically informed strategies to optimise their experience. This qualitative study utilises semi-structured interviews with participants recruited via gatekeepers across social prescribing services in England and Wales. A topic guide, informed by the Theoretical Domains Framework (TDF) and the COM-B model of behaviour change, was used to structure interviews. Data were analysed using Thematic Framework Analysis guided by the TDF. A total of eighteen service users participated in this research. Ten were aware of social prescribing and could provide an accurate explanation of what it is; three recognised the term but could not explain the concept; and five had no prior knowledge, despite accessing social prescribing services. Overall, awareness of social prescribing, its benefits for mental health, and service accessibility were limited and identified as key areas for improvement. Eleven theoretical domains identified related to engagement with social prescribing services for mental health. Twenty-one analytical themes were generated across the eleven domains. Of these themes, seven suggested changes to the current provision, with the remaining fourteen providing recommendations for future services. Findings advance the field of social prescribing for mental health by providing insight into the experiences and perceptions of service users, underpinned by a robust behavioural framework analysis to identify behavioural determinants.

**Supplementary Information:**

The online version contains supplementary material available at 10.1007/s10597-025-01534-0.

## Introduction

Social prescribing is a non-pharmacological, community-based intervention that supports individuals to manage their health, using community-based resources. There are many models of social prescribing in operation across the UK, with the most common model used in the National Health Service (NHS) in England defined by Husk et al. (2020) as “holistic social prescribing” p310 (referral from primary care to a link worker, who works with the person to identify community-based activities to support them) (Husk et al., 2020). However, both within and outside of the NHS, community-based organisations are offering social prescribing more holistically, where referrals come from the individual themselves or a range of referrers (for example, a general practitioner, nurse, pharmacist, welfare advisor, or social worker). In all cases, a link worker uses the principles of shared decision-making (person-centred care) to support individuals on their most important needs (Cooper et al., 2023). Social prescribing, specifically for mental health, is typically delivered in the same way as for other conditions, but the initial referral into the service is based on a mental health need. While individuals could have needs that are predominantly physical or mental health-related, social prescribing provides links to physical (e.g., exercise groups), mental (e.g., anxiety), and social (e.g., housing, financial) health support, providing a holistic, person-centred approach (Kiely et al., 2022). 

Social prescribing is centred on supporting the individual; therefore, it is important that service users’ voices are heard in research. Research reporting on service users’ experiences has outlined the potential benefits of social prescribing to mental health management (Cooper et al., 2023), social connectedness (Cooper et al., 2022), and the management of long-term conditions (Pescheny et al., 2020). A 2023 systematic review looking at service user experience highlighted that person-centred care was critical to delivery and that social prescribing should provide the environment for personal change (Cooper et al., 2023). Service users are also aware of the challenges of social prescribing interventions, supporting those in the most disadvantaged communities where equity of provision is limited (Pollard et al., 2023). Research has reported on the impact social prescribing can have on an individual’s behaviour, but has failed to use a theoretical underpinning to ensure all determinants of behaviour are considered. Applying a behavioural change approach to social prescribing would provide theoretical grounding and could be used to inform the future development and optimisation of social prescribing services. If a goal for social prescribing is to provide support for mental health, underpinning social prescribing with behaviour change theory could inevitably lead to better outcomes (Cooper et al., 2023). 

Research exploring the use of behavioural theory to underpin social prescribing has specifically focused on behaviour change techniques, such as social support and comparison of outcomes (Cooper et al., 2022). It has also been suggested there could be a new technique, ‘connecting with community-based opportunity’ (defined as “*connecting the person with a community-based opportunity to facilitate performance of the desired behaviour via a direct or indirect route and using signposting*,* prescription*,* referral or a combination of these methods*”p122) which is directly linked to social prescribing (Cunningham et al., 2022). However, additional research is required to establish whether these techniques are effective for creating behaviour change and to identify an optimal theory to develop social prescribing interventions.

This research aims to explore service user experiences and perspectives of social prescribing for mental health and to develop theoretically informed strategies to optimise their experience.

## Methods

### Design

A qualitative study, utilising semi-structured interviews, was conducted to address the research aim. Ethical approval for this research was granted by Teesside University, Faculty of Health and Life Sciences Ethics Committee (Reference: 2021Jan1710Cooper).

### Research Team

Two members of the research team were PhD candidates with post-graduate qualifications (MSc) in health psychology (MC, KA). Three members (LA, DF, JS) have PhDs, with two (DF and LA) registered as practitioner health psychologists with the UK Health and Care Professions Council. All members of the research team had undertaken training in qualitative research methods, and three members have extensive experience in the conduct of qualitative research (LA, DF, JS).

### Participants and Recruitment

Individuals were eligible to participate if they were aged 18 years or older and met one of the following four criteria:

(i) Engaged with social prescribing services in the past 12 months (past service users),
(ii) Accessing a social prescribing service (current service users),
(iii) Engaged with a mental health organisation in the voluntary or charitable sector or had done so in the past 12 months (potential service users),
(iv) Self-report symptoms of anxiety and depression and have not accessed a service in the third sector (eligible service users).

Participants were recruited using six gatekeepers, all social prescribing link workers, based in different social prescribing services across England and Wales. The services all accepted self or primary care referrals and provided person-centred support (based on the individual needs and preferences) in person. Recruitment materials were posted to social media (X and Facebook), including newsletters, produced by services, networks, and third-sector organisations that provide social prescribing services. If service users were interested in the research, they were asked to contact a member of the research team directly by email. Participants were offered a voucher to the value of £30 as remuneration for their time.

## Data Collection

Interview topic guides (supplementary material 1) were developed iteratively and used to guide upcoming interviews. They were primarily structured around the three stages of social prescribing: (i) referral, (ii) SPSP connection, and (iii) activity and support.

There were two topic guides, one directed at those participants who were involved with social prescribing before (past, current, and potential service users), and one guide with questions directed towards those who had not accessed social prescribing (eligible and potential service users).

Finally, the topic guide included revised questions from the COM-B questionnaire (Spall, 1998) to provide a theory-informed approach that was directly linked to the Theoretical Domains Framework (TDF) (Cane et al., 2012).

The COM-B questionnaire items are based on the COM-B model of behaviour change, where C = capability (i.e. does a person feel capable of performing the behaviour), O = opportunity (i.e. does the person feel they have the chance or support to perform the behaviour), M = motivation (i.e. does the person feel they should or want to perform the behaviour), and B = behaviour. The topic guide used these aspects of exploring behavioural intention, which can be seen in Sect. 2 of each topic guide. The COM-B questions covered all aspects of the COM-B model and ensured that participants were asked about their capabilities, opportunities, and motivations that influence social prescribing behaviour (Spall, 1998). The answers to these questions were, in the first instance, closed responses (yes/no), but were followed-up by asking participants to explain their answers and provide more context for their reasoning. The COM-B questions were tailored to social prescribing by providing examples related to the service or mental health specifically. For example, “How important do you believe the following is about social prescribing services: to know more about why it is important (capability); having the time to engage (opportunity); feeling that you want to do it enough (motivation)?”

The COM-B components were directly mapped to each domain of the TDF, a 14-domain framework that links to components of the COM-B as illustrated in Table 1. The TDF was created by an expert consensus of the theoretical domains from 83 theories. Each domain of the TDF has also been developed into a question, which supported topic guide development (Michie et al., 2014).

**Table 1:** COM-B model mapped to the theoretical domains framework

COM-B	TDF domain
Capabilities	Knowledge
	Skills
	Memory, attention and decision processes
	Behavioural Regulation
Motivation	Social/professional role and identity
	Beliefs about capabilities
	Optimism
	Beliefs about consequences
	Intentions
	Goals
	Reinforcement
	Emotions
Opportunity	Social influences
	Environmental context and resources

Interviews were conducted via Microsoft Teams or by telephone, depending on participant preference. Interviews were audio recorded and transcribed verbatim. All personally identifiable information from the transcripts was anonymised.

### Data Analysis

Interview data were analysed using the Theoretical Domains Framework (TDF) (Cane et al., 2012, Hallsworth et al., 2020) followed by Thematic (Framework) Analysis (TFA)(Ritchie et al., 2013, Braun & Clarke, 2006) within each TDF domain. Data were analysed following five key stages: familiarisation, identifying a thematic framework, indexing, charting, and mapping and interpretation. This approach enabled systematic comparison and synthesis of themes across cases while maintaining transparency and analytical rigor. All stages of data analysis were completed by two researchers (MC/KA) independently, with subsequent peer debriefing with the wider research team (DF/LA/JS). Initially, transcripts were mapped according to the TDF, supporting structured exploration of patterns and associations relevant to the research objectives. Once all mapping was agreed, TFA was used to index data within each theoretical domain, independently. The term ‘analytical theme’ is used to report the higher-order themes created from data. Each theme was created by identifying content and significant data patterns, before refining and interpreting the meaning within the themes. Within each higher-order theme, there are TDF categories and sub-analytical themes. Each theme was given a label and a description based on the interpretation of collective meaning.

A range of methods were used to maximise the trustworthiness (confidence in methodology, data, and interpretation) of this research. Interview transcripts were independently mapped by two researchers (MC, KA), who created themes independently (Adams et al., 2016; Janesick & Peer, 2015). All members of the research team reviewed each stage of the analysis (Hadi & José Closs, 2016). All themes were presented with a rich description and supporting direct quotes from participants were used to enhance transparency (Spall, 1998). Finally, the use of TFA and the TDF is an interpretative process whereby themes are created based on the collective account that accurately reflects the voice of the participant. This enables the robustness of any conclusions to be tested through discussion, which serves to enhance overall trustworthiness (Adams et al., 2016; Janesick & Peer, 2015; Hadi & José Closs, 2016; Spall, 1998). 

## Results

Eighteen participants agreed to take part in this research. Ten participants reported awareness of social prescribing and could provide an accurate explanation of what social prescribing is. Three participants were familiar with the term social prescribing but could not explain the concept. Five participants reported no knowledge of social prescribing, despite being recruited from a social prescribing service.

Eight participants had previously or were currently involved with a social prescribing service (past, current, and potential service users). Ten participants reported never having been involved with a social prescribing (or similar) service to their knowledge; however, felt they would benefit from a service like social prescribing following the description of what it was (eligible service users). Participants were never explicitly asked about their mental health difficulties, but regularly reported experiencing symptoms of anxiety, depression, social isolation, or loneliness.

### Experiences and Perspectives of Social Prescribing Services

Eleven theoretical domains were mapped related to engagement with social prescribing services for mental health. They were knowledge; skills; memory, attention, and decision processes; beliefs about capabilities; optimism; beliefs about consequences; intentions; goals; emotion; social influences; and environmental context and resources.

**Fig. 1:**
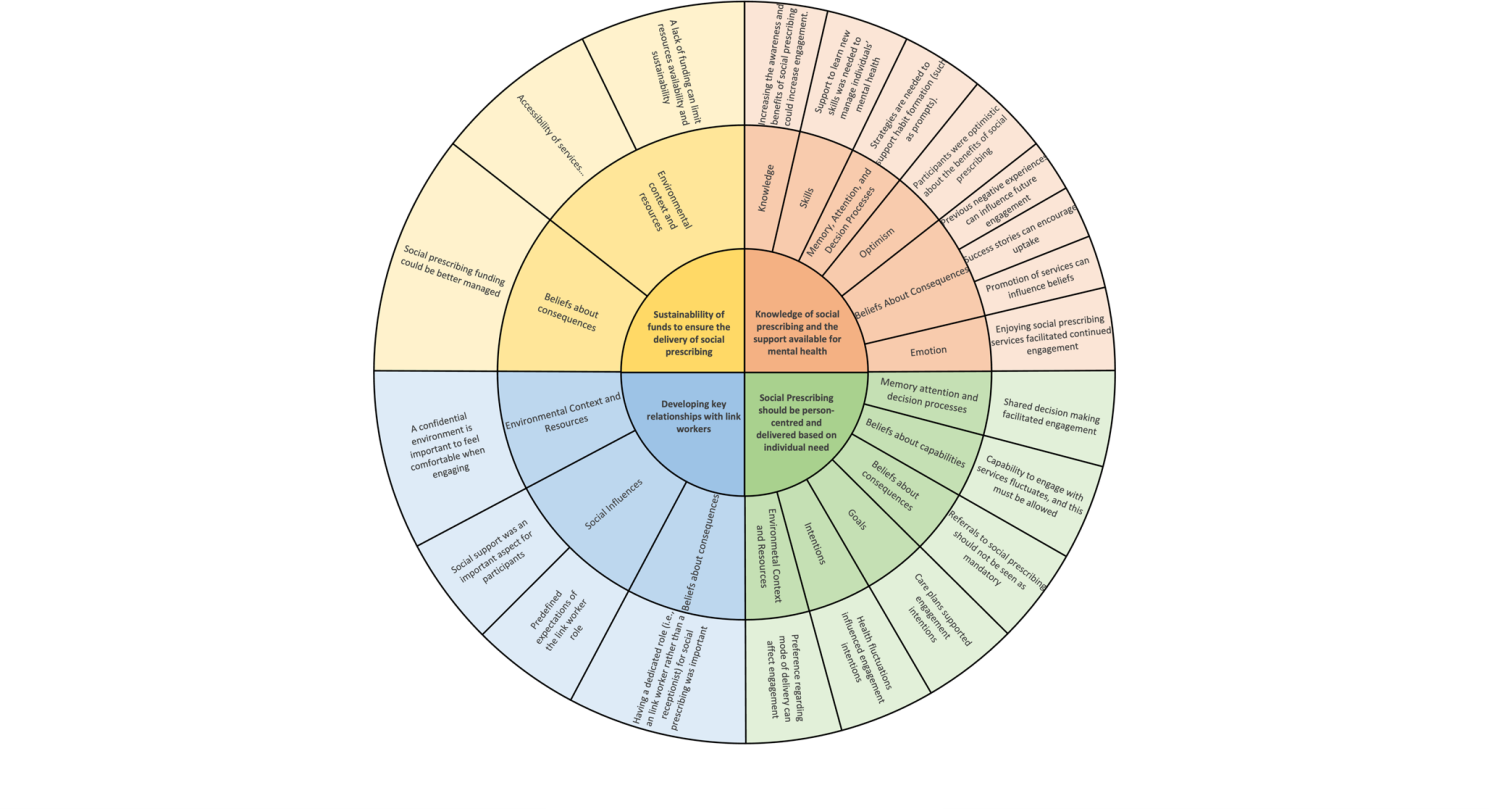
Summary of the findings linked to TDF Domains. Innermost Circle – Higher Order Themes, 2nd Innermost Circle – Theoretical Domains, Outside Circle – Analytical Themes

Twenty-one analytical themes were generated across the eleven domains. Of these themes, seven suggested changes to the current provision, with the remaining fourteen providing recommendations for future services. Figure 1 presents a summary of the findings in accordance with the TDF domains.

### Knowledge of Social Prescribing and the Support Available for Mental Health

This higher-order theme was captured within six TDF domains. The theme details the need to have the appropriate knowledge and optimism about the benefits of social prescribing, skills to manage mental health, strategies to support change, and enjoyment of activities.

#### Knowledge: Increasing Public Awareness of the Benefits of Social Prescribing Could Increase Engagement

Participants felt there was a lack of understanding about what social prescribing is and that *“most people would have never heard of that word before*” (P10). Without people knowing what it is, participants felt there could be a hesitancy to engage. There was a reported need for more information about what social prescribing is and how engaging with a social prescribing service could benefit their mental health, wanting to *“know more about what would be involved there*,* what kind of support they would provide*,* and how long-term the support [would be]*” (P11). Participants felt that increasing knowledge of social prescribing and giving people time to understand the support on offer would increase the likelihood of engagement in the future.

#### Skills: Support to Learn New Mental Health Management Skills

Participants reported the need for support to learn new skills for the management of their mental health. They spoke about the difficulties they faced managing their mental health and expressed a desire to receive support from social prescribing. “*If it could give you the skills to be able to deal with it*,* that would be brilliant”* (P2). For social prescribing to have a lasting effect, participants were aware that the acquisition of skills would enable them to manage their mental health. One participant reported,

*“If you don’t teach somebody skills in how to manage their own mental health*,* then if something happens in the future where their mental health is affected again*,* they won’t know how to stop the cycle”* (P6).

Empowering people through skills development within social prescribing was a positive motivator for engagement.

#### Memory, Attention, and Decision Processes: Strategies are Needed to Support Habit Formation (Such as Prompts)

Creating long-term behaviour change (referred to as ‘habits’) was considered an important aspect of management of mental health, with habits being both positive and negative influences. Participants felt that social prescribing should be able to help remove detrimental habits and provide strategies to build new, more positive habits. Participants were aware that developing a new habit was “*an effort”*, but once in place, “*it makes everything so much easier”* (P13). There was also an understanding that habits take time to develop and that it would involve a process of working with a link worker overtime.

#### Optimism: Optimistic About the Benefits of Social Prescribing

Participants viewed social prescribing as a positive support in their lives, where it could “*really fill the gaps in mental health support”* (P11). Participants felt strongly about how social prescribing could support those with mental health difficulties and provide “*a soft-touch approach to mental health”* (P11).

Participants liked how there were different options available for a social prescription and likened it to when “*doctors have more than one pill to give…you get different pills for different things”* (P18). They were all optimistic about the benefits they could potentially receive from social prescribing to support mental health (including those who were unclear of the nature at the beginning of the interview).

#### Beliefs About Consequences: Previous Negative Experiences can Influence Future Engagement

Participants reported having received a range of different help (statutory and non-statutory) to support their mental health, with some negative experiences (e.g., poor relationships with providers or feeling blamed for poor outcomes). Participants spoke about how social prescribing should be different and *“not penalising people for not having it work immediately*,* or all the time*” (P8). Having a bad experience with a service not only impacts participants’ beliefs about that service but also affects accessing other services (e.g., one negative experience will mean all subsequent ones will be the same). This can create barriers to engagement and setbacks: “*that stops you accessing other places because you think you’re going to have the same experience again*” (P4).

#### Beliefs About Consequences: Success Stories Could Encourage Uptake

Participants felt they would benefit from hearing about how social prescribing has helped others when deciding whether to engage *“because [I] don’t want to spend time doing something if it’s not going to be of benefit”* (P6). They wanted access to services they felt would be of most benefit to them and the outcomes they wanted to achieve. If there are relatable case studies (based on need, preferences, or characteristics), this could increase the likelihood that people would engage with social prescribing:

“*It’s a fairly new thing*,* and if people haven’t heard of it*,* you know*,* then they might see it as like*,* oh*,* have other people done this?”* (P9).

#### Beliefs About Consequences: How Services are Presented can Influence Beliefs

How social prescribing services are presented can influence beliefs, and as such, engagement. Primary care is a core referral pathway, and if participants felt their questions were unanswered, it was reported to have a detrimental effect on uptake. Participants felt people could be better informed about what social prescribing could offer and the benefits of supporting their health.

“*The GP might not have enough time to try and fully explain everything and I guess …less people might take it up less often if they haven’t been able to spend more time thinking about it*” (P6).

#### Emotion: Enjoyment Facilitated Continued Engagement

Participants wanted to feel a sense of joy or fun with social prescribing support, as they felt this would lead to increased motivation. *“If you don’t enjoy it*,* or you don’t have a sense of satisfaction then you’re less likely to engage”* (P6). This was essential and was felt to be something that would also support longer-term engagement. “*Very important to have a good experience*,* because if you had a bad one you kind of mentally lock yourself out of going back”* (P17).

### Social Prescribing Should be Person-Centred and delivered based on individual Need

This highest-order theme was captured in six TDF domains. The theme details the need to ensure social prescribing is needs-led, person-centred, and flexible to ensure engagement.

#### Memory, Attention, and Decision Processes: Shared Decision-Making Facilitated Engagement

For many participants, social prescribing provided them with a link worker who they felt spent the time to support the choice in what their care looked like and gave decision-making power to them. Participants felt this was hugely positive and beneficial to the way they would interact with a service.

#### Beliefs About Capabilities: The Capability to Engage with Services Fluctuates, and This Must be Allowed

Participants reported how fluctuations in their mental health would affect their capability to regularly engage with services, and this should be acknowledged by link workers. “ [It is] *Really hard and sometimes you might be halfway down that hole until you think*,* ‘well*,* actually*,* I do need some help’”* (P1). Participants reported wanting social prescribing to acknowledge fluctuations in health and support them to engage when they needed it. Participants’ capability to engage in support was also dependent on the medication they were taking. Allowing individuals to engage with a service on their terms and supporting them to be capable of engaging was reported to be important to participants. They reported:

*“To get to a point with treatment where [people in need] function sufficiently that they can benefit from building all the mental well-being health side of it from social prescribing*” (P5).

#### Beliefs About Consequences: Referrals to Social Prescribing Should not be Mandatory

Participants expressed the view that engagement with social prescribing should be a choice; for example, in the much the same as if they were offered medication. Participants felt that social prescribing should not be ‘pushed’ onto them and needs to be an option alongside medical and psychological therapies, including self-help’; otherwise it could be damaging to their motivation to attend a social prescribing service. Participants reported not wanting it to be “*a carrot and stick approach”* (P11). They expressed that the more they were coerced to do something, the less inclined they would be to do it. *“If you’re feeling like you’re being guilted into it or you should do it*,* then you’re not really doing it because you want* to” (P11).

#### Goals: Care Plans Supported Engagement Intentions

Participants felt that having goals and plans was important, and participants liked how they could use a plan to help:

“*Transition out of services and back into your own life*” and “*having things written down to refer back to and get you back on track if you have a lapse or relapse is essential”* (P11).

Participants wanted a plan for how they would engage with services to support their mental health. Ideally, this plan would be written down or given to the service user, so they can refer to it at any time. Despite the desire for plans, it was emphasised that plans that were ‘*too rigid… wouldn’t work out’* (P14).

#### Intentions:Health Fluctuations Influenced Engagement Intentions

Participants’ intentions to engage with a service often fluctuated depending on their health (linked to the previous domain, Beliefs About Capabilities). Participants reported that social prescribing should look to encourage and motivate people to engage at a time, they believe, is best for them. Participants acknowledged that:

“*If you’re motivated to change the circumstances that you’re in*,* if you can’t recognise that*,* there’s a problem if you [are] wanting to make a difference*,* then you’re not going to do it”* (P11).

#### Environmental Context and Resources: Preferences Regarding Mode of Delivery Can Impact Engagement

Participants were interviewed post the COVID-19 pandemic, and for those who had been involved with services, they reflected on what had subsequently changed. Participants reflected on how their preferences had changed and were more accepting of “*a Zoom call or maybe a telephone call”* (P10). This would allow them to gain support when accessing support in person was difficult.

Despite the acceptance of digital methods of engagement, there was a universal preference for in-person engagement. Participants believed that the link worker could get “*a bit more of an idea of the person’s physical ability*,* face-to-face cognitive*,* psychological state”* (P5). They also felt that *“human contact*,* if at all possible*,* is pretty vital*” (P5) for improving mental health, and it created a more personal connection.

Regarding the duration of time spent with the link worker, it should not be limited to several sessions, and instead, let the link worker use their professional judgement. This was reflective of the belief that *“it can often take a very long time to get someone back when they have this long problem*” (P17). Participants wanted to feel they were being supported and not rushed through a service, which would negatively impact their mental health.

### Developing Key Relationships With Link Workers

This highest-order theme was captured in three TDF domains. The theme details the need for a confidential workspace where social support can be provided to the individual. It also captures the role of the link worker in setting expectations of what social prescribing can offer and the boundaries of the role.

#### Beliefs about Consequences: Having a Dedicated Link Worker Role was Important

Having a dedicated role, such as a link worker, to deliver social prescribing services was a core element of social prescribing for participants. They felt it was “*probably way more beneficial to have someone whose actual job it is to just do that*” (P8). Participants liked the:

“*Idea of someone [who it] is their expertise*,* because I think with GPs and other healthcare professionals*,* they [can’t] because of everything else they have to do*,* they might not know of all the services*,* or they might not be so personal about it”* (P9).

Participants believed that being able to promote choice in a person-centred (shared decision-making) approach was best achieved with a dedicated person in a link worker role.

#### Social Influences: Predefined Expectations of the Link Worker Role Were Needed

Participants felt an openness with link workers, where they were listened to and their beliefs were respected. This subsequently helped them to discuss what mattered to them. Participants who had not met with a link worker commented on how they would expect to meet with someone who had some “*life experience”* and if they were going

“*to meet with somebody and they look liked they had their life all sorted and they were like a lot younger than me*,* very professional that would be a negative”* (P15).

Participants spoke about how they wanted to have trust and confidentiality from link workers. They felt:

*“Particularly for mental health*,* it’s so important to really trust the person that’s supporting you and to get a good relationship with them*,* so that you can feel more comfortable taking up the recommendations that they’re making”* (P11).

Building a relationship was about developing a level of social support they would not normally receive from other health professionals, which made social prescribing stand out. Participants wanted regular contact with their link worker and felt link workers should provide a follow-up contact after they had left the service. This would help support re-engagement if the person felt it was necessary and had a pre-established relationship with the link worker.

#### Social Influences: Social Support was an Important Aspect for Participants

Participants stated motivation to engage with social prescribing was greater with peers who have their own lived experiences of mental health. Participants reflected on how it was:

“*Really important to know that you’re not on your own and that other people are on a similar journey”* (P13).

There was a desire to see how peers had benefited from social prescribing and to learn from others who had been supported. In addition, participants felt there should not be an expectation of social support from family and friends, with many preferring to receive support from only others with lived experience. They also felt there needed to be better resource management (funding) to benefit more people, “*a lot of GP surgeries have had this funding and they’re not sure how to implement it”* (P15).

#### Environmental Context and Resources: Accessibility of Social Prescribing may Affect Engagement

The accessibility of social prescribing was essential to ensuring engagement. Impacting accessibility were the geographical location and appointment availability. “*Personally*,* I work full-time*,* Monday to Friday*,* so it can be quite hard to get appointments”* (P11). Others felt the lack of timely support affected their engagement:

“*Things like that you can procrastinate over such a long time that when you actually go and reach out for help then if it takes too long… you know*,* it can be time sensitive”* (P14).

#### Environmental Context and Resources: a Lack of Funding can Limit Resource Availability and Sustainability

Participants felt there was a need for additional funds to support social prescribing delivery and the resources available to link workers. They wanted social prescribing to be available to them in the future and felt that: *“Nothing that gets the funding it needs or deserves. Without the money in to do the job*,* it isn’t going to work”* (P18).

Participants reported the need for more accessible resources and more options to develop skills to support their own mental health, which they felt was currently prevented by a lack of funding.

## Discussion

This research explored service user experiences and perspectives of social prescribing for mental health and to develop theoretically informed strategies to optimise their experience. Advancing the field further, this study involved combining the TDF with the COM-B model to map factors influencing uptake and engagement onto the components of the COM-B (Spall, 1998). Table 2 highlights the themes and subsequent domains that are mapped to specific components of the COM-B model. This provides a grounding in behavioural theory that is often lacking in social prescribing research,2 providing insights into the behavioural influences on future optimisation of social prescribing services. Overall, this research identified the capability, opportunities, and motivational barriers to people engaging in social prescribing services for mental health.

**Table 2:** Triangulation of COM-B, TDF, and Themes

COM-B	TDF Domain	Analytical Theme (Higher Order Theme)
Capabilities	Knowledge	Increasing public awareness of the benefits of social prescribing could increase engagement (1)
	Skills	Support to learn new mental health management skills (1)
	Memory, attention and decision processes	Strategies are needed to support habit formation (such as prompts)(1) Shared decision-making facilitated engagement (2)
Motivation	Beliefs about capabilities	The capability to engage with services fluctuates, and this must be allowed (2)
	Optimism	Optimistic about the benefits of social prescribing (1)
	Beliefs about consequences	Previous negative experiences can influence future engagement (1) Success stories could encourage uptake (1) How services are promoted can influence beliefs (1) Referrals to social prescribing should not be mandatory (2) Having a dedicated link worker role was important (3)
	Intentions	Health fluctuations influenced engagement intentions (2)
	Goals	Care plans supported engagement intentions (2)
	Emotions	Enjoyment facilitated continued engagement (1)
Opportunity	Social influences	Predefined expectations of the link worker role were needed (3) Social support was an important aspect for participants (3)
	Environmental context and resources	Preferences regarding the mode of delivery can impact engagement (2) Accessibility of social prescribing may affect engagement (3) A lack of funding can limit resource availability and sustainability (3)

Overall, this research highlighted a lack of knowledge about social prescribing (capability), even among those who currently engage with social prescribing services. There was also a lack of knowledge about the benefits social prescribing can offer (capability), particularly around developing skills to manage mental health. The accessibility of services was also identified as an area requiring improvement (opportunity).

Critically, this research provides a behavioural understanding of the experiences and perceptions of service users, which is critically important to understanding uptake and continued engagement with social prescribing services. Interview data were mapped using the TDF, which linked participant responses to eleven theoretical domains (i.e., knowledge; skills; memory, attention, and decision processes; beliefs about capabilities; optimism; beliefs about consequences; intentions; goals; emotion; social influences; and environmental context and resources). The TDF domains identified highlights the behavioural complexity of long-term engagement with social prescribing and where optimisation of behavioural theory can actors impacting mental health.

This is particularly apparent with the overlap between analytical themes across TDF domains. For example, the issue regarding the use and allocation of funding was mapped to beliefs about consequences and environmental context and resources. These two domains provide an opportunity to explore how funding affects how service users perceive social prescribing (opportunity), the sustainability of resources available (opportunity), and the potential to provide more options to individuals accessing services (opportunity), particularly to facilitate self-management of mental health.

The impact of mental health fluctuations was a consistent finding and mapped to TDF domains beliefs about capabilities and intention (motivation). These domains articulate how mental health fluctuations influence the service users’ capability to engage with social prescribing and their overall motivation to participate in ongoing support. This indicates the need to have services that are sensitive to this issue and can adapt and be sufficiently flexible to support the needs of the service user. A service that predicts changes to capability and motivation and provide the right kind of support at the right time is optimal in this situation. Using this example, the TDF has provided a deeper understanding of the behavioural factors influencing a person’s ability and willingness to engage with social prescribing and could assist in advancing the field.

The lack of public awareness and knowledge about social prescribing is consistent with the findings reported in previous research (Bild & Pachana, 2022; Khan et al., 2022), including amongst healthcare professionals (capability) (Scott et al., 2021). Research has reported that a lack of clear and consistent language can lead to confusion over referrals and service users being unaware they are part of a social prescribing pathway (Carnes et al., 2017). This lack of consistent language is also an issue for the job title and definition of the link worker role, where there are a multitude of titles currently in existence, for example, community linker and social prescriber (Carnes et al., 2017). This ambiguity and the lack of knowledge reported by participants in this study highlights a key area where future practice could improve.

Similarly, there was a lack of awareness about the support social prescribing could offer individuals struggling with their mental health (opportunity). This highlights that more could be done to promote social prescribing, for example, by using peer support or success stories. Currently, in practice, this can be seen with the promotion of success stories within the UK media (Hanlon et al., 2021). The National Social Prescribing Academy has already commenced work to impact public perception of social prescribing services using social media and podcasts, however this has yet to be evaluated (Khan et al., 2022). Future social prescribing research and practice should encourage publication and promotion of the potential benefits of social prescribing to a wide range of audiences.

Social prescribing services for mental health are grounded in person-centred care (Cooper et al., 2023). The findings of this research emphasise the need for social prescribing to continue to be person-centred and ensure decisions made about care are done so via shared decision-making with the individual (motivation). Previous research has equally stressed the importance of person-centred care within social prescribing (Afuwape et al., 2010), and concluded there is a lack of reporting around the design of person-centred support (Cooper et al., 2022). 

The findings of this research study also report on the need for service users to be actively involved in the decision-making and planning of their care, with person-centred goals, skills development, and onward referrals (capability). Adopting a person-centred approach to each of these areas has been suggested to increase confidence and capability to manage conditions (Furze, 2015), reduce mental distress and improve mental health outcomes (Afuwape et al., 2010; Kilgarriff-Foster & O’Cathain, 2015; Roberts & Windle, 2020). This was particularly highlighted in the analytical theme shared decision-making facilitated engagement, where participants expressed the desire to be actively involved in planning. Future research and practice should focus on ensuring a person-centred approach to delivery by establishing a quality framework for link worker practice.

For service users, it was important to develop a therapeutic relationship with link workers as they were seen as valued sources of social support (opportunity/motivation). The therapeutic relationship between link workers and service users was viewed as critically important for achieving positive outcomes. Wider literature has reported that boundary setting, expectation management, and fostering independence from the link worker were critical to ensuring positive outcomes for service users (Moffatt et al., 2017; Sharman et al., 2022). However, ensuring that link workers can meet the needs of service users requires both training and appropriate referrals from external services, as negative experiences can impact a service user’s perspectives of social prescribing (Sundaram et al., 2012). 

Collectively, these findings highlight the practical environmental context and resources required to maximise uptake, engagement, and support sustainable social prescribing services for mental health (opportunity). This research study captures the awareness of service users of the increased demand for mental health support. Following the COVID-19 pandemic, there have been economic implications that have increased poor health, including mental health, and exacerbated health inequalities (Younan et al., 2020). The pandemic provided a pivot for social prescribing to offer a digital delivery (increasing reach to those who may not have previously engaged in face-to-face support), but the impact of digital exclusion and inequity has yet to be explored (Morris et al., 2022). There was a strong belief among participants of this research that the rising demand for social prescribing could lead to the overwhelming of resources in the community (opportunity). Service users believed that if social prescribing was not appropriately managed, with clear plans and sustainable funding, this would lead to issues with capacity (motivation). This finding is consistent with findings reported in a 2021 systematic review (Brown et al., 2021). 

This research presents a series of suggestions and recommendations arising from the experiences and perceptions of service users of social prescribing services to support their mental health. Future research and practice of social prescribing for mental health should consider these recommendations and the behavioural insight generated to promote uptake, engagement, and overall experience of social prescribing to increase the likelihood of positive changes to mental health.

## Strengths and Limitations

This research provided a detailed account of the experiences and perspectives of social prescribing service users in relation to social prescribing services for mental health. The approach to data analysis, using the TDF and TFA provided an important understanding of key behavioural determinants of uptake and engagement with social prescribing services. Critically, this helps to advance the field of social prescribing by providing a using health behaviour change framework to optimise services in the future. Applying this method enabled a more detailed insight into the key theoretical behavioural constructs within social prescribing and addresses previous research conclusions highlighting the lack of theoretical application (Cooper et al., 2022). 

However, the approach to recruitment (the use of gatekeepers and social media) may have created an element of selection bias (Pocock et al., 2021; Cooper et al., 2024). There has been much debate over the representativeness of samples when using social media as a tool for recruitment (Cooper et al., 2024; González-Bailón et al., 2014). Therefore, it is likely that the sample of participants recruited does not adequately represent the wider population of service users, despite aiming to reach maximal variation. There were no measures of sample diversity using demographic data (although the geographical representation of social prescribing services was diverse in the sample). Therefore, no conclusions can be made about how the representative the sample included is compared to the social prescribing population.

## Conclusions

The findings of this qualitative research study advance the field of social prescribing for mental health by providing insight into the experiences and perceptions of service users and linking those to a behaviour change framework to identify key behavioural determinants. Four main recommendations arise from this research.

1. There needs to be increased public awareness of what social prescribing is and the benefits that can be derived for mental health.
2. Social prescribing services should be delivered in line with person-centred care, using shared decision-making.
3. Social prescribing should ensure that link workers are trained in the development of therapeutic relationships with service users.
4. Research should directly inform the development of policy for social prescribing, ensuring sustainability of funding and allocation of resources.

## Supplementary Information

Below is the link to the electronic supplementary material.

Supplementary Material 1 (DOCX 24.5 KB)

## Data Availability

No datasets were generated or analysed during the current study.
