# Supplementary material for: Service User Experiences and Perspectives of Social Prescribing Services for Mental Health
Source: Community Ment Health J. 2025 Oct 21;62(3):405–16. doi: 10.1007/s10597-025-01534-0 (PMC12963173; doi:10.1007/s10597-025-01534-0)
Supplement: Supplementary file 1 — Supplementary Material 1 (DOCX 24.5 KB) [file 10597_2025_1534_MOESM1_ESM.docx]

Supplementary Material 1: Topic Guide

1. Have you heard of social prescribing before, and if you have could you please tell me what it is?
   1. IF YES to Q1
      1. Have you accessed a social prescribing service before?
         1. IF YES move to table side A
         2. IF NO move to table side B Part 2
   2. IF NO to Q1 move to table side B Part 1

| Table Side A | Table Side B |
| --- | --- |
| *replace social prescribing with the name of the service they have been recruited from throughout. | PART 1 |
|  |  |
| When did you first accesses the [social prescribing]* service? | Okay so social prescribing is a way of connecting people with the help and support they need from their community. This could involve anything from help with housing or financial problems to getting involved with local community groups such as gardening or social groups. Usually, your GP will refer you to a link worker, who is someone who knows what groups and support are out there to help. Usually once you have been referred a link worker, they will agree to meet you and discuss what matters most to you and how they can help. This doesn’t have to be the main reason for why you attended the GP in the first place, but whatever you feel is the most important. |
| How long have you been with the current service or the last service you accessed? | **Is this something you have heard of before?**  IF YES   - Has it been called something else? - Where have you heard of it? - Has anything similar ever been offered to you?   IF NO   - Does that sound like something that would be helpful to you? - If you were offered a social prescription, would you be interested in it? |
| When was the last time you used the service? |  |
| Region of service that you are in? |  |
|  |  |
| **Pre-Pandemic** | **PART 2** |
| **How did you become involved in the** [social prescribing]* **service?**  Prompt:  What were your expectations?   - What did you know about [social prescribing]*?   Did you feel that [social prescribing]* could help and was right for you?   - Why and what was the reasoning behind it   What services did you access or use?   - What appealed to you about those in particular - How were they identified to you? - Did you benefit from them? - How often did you use them? | **If your GP offered you a social prescription for a mental health issues, would you agree to attend an initial appointment with a link worker?**  If yes/ no – please could you explain a little more?  Link worker   - What would you expect to happen in an initial meeting? - What would you want to gain from the initial meeting? - Do you think a link worker is necessary?   Would you be happy to self-refer to an organisation for a social prescription if you were given the option?  How long would you be willing to wait before your initial meeting with a link worker/ receive a social prescription? |
| **What were the main barriers and facilitators that you faced when using the service?**  Prompt:  Why were they barriers/ facilitators   - Was there anything done to try and address these - Was there any additional emphasis placed on these? - What could have been done to change them?   Link worker/facilitator   - Useful and helpful? | **What would be your preference for where and how to meet with a link worker?**  Prompt   - In person (face to face as a GP surgery or elsewhere? - Telephone (audio based) - Online video calls? - If your preferred option is in person (face to face), would you prefer to go to the link workers or for the link worker to come to you at a location of your choice? |
| **Post- Pandemic** |  |
| **Did the Covid-19 pandemic affect your use of service in any way?**  If YES- how was it affected, what impact did this have on the way you use the service?  If NO – how has the service-maintained support and was there anything additionally offered to you?  Prompt:  Change in need   - Was there a change in the requirement of what you needed? - If yes what was the change to - Did your use of the service change?   Adjustments made   - Was there anything that changed about the service over the course of the pandemic? - If yes what changed and how did that impact you - If no did that impact you   Was there any changed to the way that the service was delivered?   - If yes – did if work for you and how did you feel about it? - If no – did that impact how you accessed the service | **What type of support or help might you like to receive from a social prescription?**  Prompt:   - Housing, financial/ debt - Isolation and loneliness - Physical health - Mental health - General wellbeing   What type of activates might you wish to attend?   - Vocational courses - Activities such as organised nature walks - Arts based groups - Others? |
| **Do you think the service needs to change for a post -pandemic service user?**  Prompt  Provision of services   - Anything that is additionally required - Anything that should be removed   Scope of the service   - What should be offered or suggestions for what could additional be offered   Changes to delivery style   - Online based - group vs individual based   Do you think services should be planning for future lockdowns and pandemics? | **What types of things might get in the way (barriers) or encourage you (facilitators) to attend a charitable or voluntary organisation like this for your mental health?**  **Prompt:**  why do you think that?  Is there anything that could be done to change that?  Does it sound like something you would be interested in? |
|  | **Do you think social prescribing is something that could help others living with mental health conditions?**  Prompt:  Why do you think that?  What are the key things people should know to let them know what social prescribing is?  Do you think social prescribing is something that would have helped you or others during the lockdown periods in the pandemic? |
|  |  |

FOR ALL

***BCW Analysis Questions***

1. **I am going to read out several statements**. For each one I will ask you to state how important you think it is in relation to the SP intervention you have received or will receive in the future. For those statements that you do think are important for you, it would be useful for us to know why.

The answers you and others give to these questions will help to develop more effective SP interventions in the future.

Capability Prompts:

- 1. **To know more about why it is important** – e.g., have a better explanation of the benefits of engaging with a SP.
  2. **Knowing more about how to do it** – e.g., have a better explanation of how to use SP services to get the best out of them.
  3. **Having better Physical or mental skills** – e.g., to develop new skills through the SP intervention that helps you to effectively manage your own mental health.
  4. **Being able to overcome physical or mental limitations** – e.g., being able to continue working with SP services when you are feeling tired or having a bad day mentally.
  5. **Having physical or mental stamina** – e.g., developing or increasing the stamina you have to help keep you engage with a SP service.

Opportunity Prompts:

- 1. **Having the time**– e.g., having more time to focus on your mental health.
  2. **Having the necessary resources** – e.g., groups, information, helplines available to you to help you improve your mental health and wellbeing.
  3. **Having peer support** – e.g., being part of a group of people who are also going through a similar journey to help you on your way.
  4. **Having reminders about what to do**– e.g., having reminders at important times to prompt you to use what you have learned from the SP intervention.
  5. **Having support from others**– e.g., from those who maybe part of your family or friendship group.

Motivation Prompts:

- 1. **Feel that you want to do it enough** – e.g., having a sense of satisfaction and enjoyment from using SP services.
  2. **Feel that you need to do it enough** – e.g., care about the negative consequences of not using SP services.
  3. **Believe that it would be a good thing**– e.g., having a strong sense that you should use SP services to help improve your mental health and wellbeing.
  4. **Develop better plans for doing** it – e.g., having clear and well-developed plans for improving your mental health and wellbeing.
  5. **Having better habits–** e.g., getting into a pattern of actively improving your mental health so you do not have to think too much about it.

Anything else

1. **We are nearing the end of the interview now. Is there anything else that you would like to mention about social prescribing or the service you are involved with?**

**Thank you for your time**
